# Integration of the Integrate, Design, Assess, and Share Framework in Developing the Environmental Health Literacy Toolkit Paraben-Free & Me: Protocol for a Randomized Controlled Trial

**DOI:** 10.2196/85474

**Published:** 2026-02-13

**Authors:** Graziella De Michino, Satveer Dhillon, Caroline Barakat

**Affiliations:** 1 Faculty of Health Sciences, Ontario Tech University, 2000 Simcoe Street North, Oshawa, ON, L1G 0C5, Canada, 1 6475159369; 2 Department of Geography and Environmental Management, University of Waterloo, Waterloo, ON, Canada

**Keywords:** educational toolkit, behavior, endocrine disruptors, knowledge translation, parabens

## Abstract

**Background:**

Endocrine-disrupting chemicals, such as parabens, are commonly found in personal care products (PCPs). Exposure to parabens is linked to several significant health risks, such as reproductive disorders, breast cancer, infertility, and hormone imbalances. Women are particularly vulnerable to these effects due to their higher use of PCPs containing parabens. Despite these risks, Canada lacks regulatory frameworks for the use of parabens in PCPs, relying instead on consumer awareness for reducing exposure. Previous studies have highlighted that many women remain unaware of parabens, exhibiting low risk perception and limited knowledge, which restricts behavior change toward safer choices.

**Objective:**

To address this gap, this project developed the Paraben-Free & Me educational toolkit using the integrate, design, assess, and share framework.

**Methods:**

Toolkit development methodology involved empathizing with target users, defining specific behaviors, grounding the data in the health belief model, ideating implementation strategies, prototyping, gathering user feedback, and building a minimum viable product.

**Results:**

The Paraben-Free & Me toolkit includes multimedia resources such as blog posts, interactive quizzes, videos, podcasts, and forums aimed at increasing knowledge, risk perceptions, and health beliefs and facilitating paraben-free behaviors.

**Conclusions:**

This toolkit shows potential to inform women about endocrine-disrupting chemicals, reduce exposures, and improve health outcomes.

## Introduction

Human exposure to endocrine-disrupting chemicals (EDCs) is widespread, as they can be found in electronics, pesticides, cosmetics, personal care products (PCPs), plastics, and other everyday products [1]. Parabens, for example, are a type of EDC commonly used as both synthetic preservatives and fragrance ingredients [2]. Women are at greater risk of exposure to parabens than men due to relatively higher PCP use, which has the potential to negatively impact their health and well-being [3-5]. For example, several studies have underscored the multifaceted impact of exposure to parabens and other EDCs, including the increased risk of pathophysiological reproductive conditions such as polycystic ovarian syndrome and endometriosis, breast cancer, infertility, and abnormal sex steroid hormone levels [2,3,6-9].

Compounding the negative impact of parabens is the lack of governmental restrictions and regulations for parabens and other EDCs in Canada. Unlike other regions, such as Europe, where there is a more robust approach to regulating EDCs [10], Canada uses a more risk-based strategy with no explicit regulation of EDCs [11]. Only products registered with Health Canada as natural health products have regulated limits on paraben concentrations [12]. By not implementing stricter regulations on parabens, the government of Canada puts the onus on the consumer to make health risk decisions.

Placing the burden on the consumer is concerning as, while there is increasing awareness of parabens and the associated negative health impacts, a large majority of women do not have a sufficient amount of knowledge about them [13-16]. To highlight this, a study by Trifunovski et al [16] conducted in Ontario, Canada, observed that women had low risk perceptions of parabens in PCPs, with 28% of participants having not previously heard of parabens. Additionally, increased knowledge, risk perceptions, and health beliefs were associated with an increase in women’s avoidance behavior [16]. Hence, if women do not possess information about parabens, it is difficult for them to implement changes to reduce their risk [13]. These findings highlight the importance of enhancing women’s knowledge and access to information, health beliefs, and risk perceptions to encourage paraben-free behaviors.

Hence, one such intervention that can be implemented to increase the awareness of parabens and, consequently, improve health outcomes is creating an educational toolkit. Educational toolkits are defined as a package of resources for knowledge translation and facilitating behavior change [17]. Toolkits typically include resources such as informational materials, checklists, guidelines, and interactive activities designed to educate and empower users. Studies have highlighted how toolkits can be used for behavior change [17,18]. A review by Barac et al [18] reported that, of the 31 included studies that evaluated toolkit effectiveness, 21 were found to be satisfactory or useful or resulted in an intention to change practice. However, there is no empirical evidence evaluating the effectiveness of a toolkit focused on parabens when used by women. To fill this gap, this paper aims to describe the methods for creating and developing an evidence-informed educational toolkit that facilitates paraben-free behavior.

## Methods

### Overview

**Figure 1.**
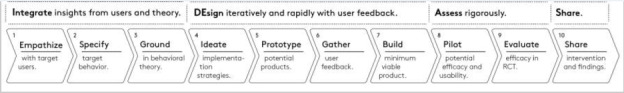
Stages of the integrate, design, assess, and share framework for developing digital interventions (reproduced from Mummah et al [19], published under Creative Commons Attribution 4.0 International License [20]). RCT: randomized controlled trial.

The educational toolkit development and methodologies were guided by the integrate, design, assess, and share (IDEAS) framework for developing digital health behavior change interventions [19]. The IDEAS framework is made up of 10 stages: empathize, specify, ground, ideate, prototype, gather, build, pilot, evaluate, and share (Figure 1 [19]). The IDEAS framework aims to produce a digital intervention that integrates behavioral theory and user feedback [19]. This framework was selected due to its focus on human-centered design and iterative process. Therefore, by grounding the development of the educational toolkit in this framework, we can ensure that it is evidence-based and its efficacy can be evaluated.

The development of this toolkit began in November 2023 and included synthesizing previous research from members of our team to help inform the project, along with partnering with the Women’s Healthy Environments Network to aid with the development of this toolkit.

### Step 1: Empathize With Target Users

The first step was to empathize with target users to understand their needs and preferences [19]. An earlier project by Trifunovski et al [16] identified 6 EDCs commonly found in personal care and household products: parabens, bisphenol A (BPA), phthalates, tetrachloroethylene, lead, and triclosan. The study aimed to understand women’s current knowledge, risk perceptions, beliefs, and avoidance behavior related to these 6 harmful chemicals. A questionnaire was administered to 200 women to analyze associations between avoidance of parabens, BPA, phthalates, tetrachloroethylene, lead, and triclosan and knowledge, risk perceptions, and health beliefs [16]. Furthermore, 10 women living in Ontario, Canada, participated in virtual interviews to understand the factors that influence purchasing and avoidance behaviors.

### Step 2: Specify the Target Behavior

The goal of step 2 was to define the target behavior to be specific and measurable [19]. Mean knowledge, health belief, risk perception, and avoidance behavior scores from a previous study by Trifunovski et al [16] were used to calculate the appropriate group mean difference between the control and intervention groups. Using a Likert scale, participants’ knowledge and awareness, health beliefs, risk perceptions, and avoidance behaviors were evaluated through a composite score for each construct. For example, lower scores indicated lower knowledge and awareness. In this study, women scored an average of 21 (SD 6) points in the avoidance behavior section (scale from 6 to 30 in the original study). This step also included reviewing the current literature on educational toolkits to ensure that our toolkit was developed using best practices to elicit behavior change. Our research team conducted a comprehensive scoping review to answer the following research question: “What elements of educational toolkits contribute to improved risk perception and positive behavior change concerning the reduction of toxicant exposure from PCPs?” [21]. For this scoping review, we searched both academic studies and nonacademic literature, such as reviews of mobile apps that focus on PCPs, to ensure that we were able to analyze the literature surrounding educational toolkits that focus on increasing risk perception and promoting behavior change regarding harmful PCPs [21]. After the completion of this scoping review, and to further understand the aspects needed in an environmental health literacy toolkit, we also conducted a comprehensive systematic review to examine the current literature on environmental health literacy toolkits [22]. For this systematic review, we had two primary objectives:

1. To identify the characteristics of existing environmental health educational toolkits related to environmental health and behavior change
2. To identify the methods and data collection tools used to evaluate the effectiveness of these educational toolkits

For our systematic review, we searched peer-reviewed published articles and gray literature, which includes research and information produced outside of traditional academic publishing sources. This includes government reports, policy documents, health service guidelines, internal organizational reports, technical briefs, professional association publications, conference abstracts, dissertations, and nongovernmental organization or charity publications. This review included gray literature to capture practical and context-specific information that is often unavailable in academic journals but is directly relevant to the development and implementation of the toolkit.

### Step 3: Ground in Behavioral Theory

The purpose of step 3 was to understand and use behavioral theories that could aid in the development of the educational toolkit [19]. The aim of this step was to ensure that each characteristic identified in steps 1 and 2 was grounded in theory. To achieve this, relevant behavior change theories were reviewed, and the health belief model (HBM) was selected as the primary framework due to its emphasis on individual perceptions, motivational determinants, and decision-making processes. The strategies and desired features found during steps 1 and 2 were evaluated for fit within the HBM, which focuses on explaining and predicting individual changes in health behavior.

### Step 4: Ideate Implementation Strategies

The team synthesized data and information that were gathered in steps 1 to 3 and developed concrete strategies to ensure that the toolkit was effective. This included brainstorming potential online platforms to build the toolkit, possible modes of delivery, and important characteristics to incorporate. Additionally, we worked on identifying key themes and mapping content to behavior change objectives. In partnership with the Women’s Healthy Environments Network, which is a nonprofit organization with the aim of educating the public and policymakers on environmental health, we ensured that our research was grounded in real-world insights.

### Step 5: Prototype Potential Product

The iterative process continued as the research team discussed the feasibility of implementing specific design ideas that were generated during step 4. We ensured that the features of the toolkit aligned with the focus of the literature, the theory, and the capabilities of the web-based platform.

### Step 6: Gather User Feedback on the Prototype

To gather user feedback, we gave our research team members (women aged 18-35 years) the opportunity to provide feedback on the first draft of our toolkit. The toolkit was emailed to the research team, and they were given 2 weeks to provide feedback.

### Step 7: Build a Minimum Viable Product

The final step was to build a functional toolkit for pilot testing. Modifications were continuously reviewed to ensure accuracy and adherence to prior steps of the process. The functionality of the toolkit was tested by the research team, with the focus being on ensuring a smooth user experience, which included a simple consistent layout, interactivity for users, and bright and accessible colors.

### Ethical Considerations

Ethics approval for this research was received from the Ontario Tech University Research Ethics Board on October 5, 2023 (17494). Informed consent to participate in this study was obtained from all participants. All participant-identifying details were omitted. No compensation was provided to the focus group, which consisted of members of the research team.

## Results

### Step 1: Empathize With Target Users

The findings suggest that women have low risk perceptions of EDCs, with 28% of participants in a previous study having not previously heard of parabens [16]. The authors of this study found positive associations between high knowledge, risk perceptions, and health beliefs and avoidance of lead, parabens, BPA, and phthalates. The qualitative paper (Trifunovski A et al, unpublished data, July 2024) concluded that social media, brand awareness, and price were all factors that impacted buying decisions for PCPs and household products. In terms of ingredients that may need to be avoided, participants avoided sulfates and fragrances. There was no mention of any other ingredients that may negatively impact health outcomes.

### Step 2: Specify the Target Behavior

This was promoting women’s paraben-free behaviors by increasing women’s knowledge and access to information, health beliefs, and risk perceptions using an educational toolkit that has been informed by the literature. Our hypothesis was that women who had access to the Paraben-Free & Me educational toolkit would exhibit a 10% improvement in paraben-free behavior (3.5 points), knowledge and access to information (3.5 points), health beliefs (2.5 points), and risk perception (2.5 points) in group mean difference scores when compared with those who did not have access to the toolkit.

Our scoping review of academic sources and users’ mobile app reviews revealed four themes of elements commonly observed in environmental health educational toolkits for facilitating behavior change: (1) toolkit accessibility and affordability, (2) simplicity of the presented information, (3) personalization of features, and (4) a clear focus on knowledge sharing [21]. This review highlighted not only the elements that are currently being featured in existing toolkits but also elements that users wish were included based on their reviews. Our systematic review highlighted the importance of toolkits being interactive, accessible, and personalized [22]. These factors were integrated into the development of our toolkit to ensure behavior change.

### Step 3: Ground in Behavioral Theory

**Figure 2.**
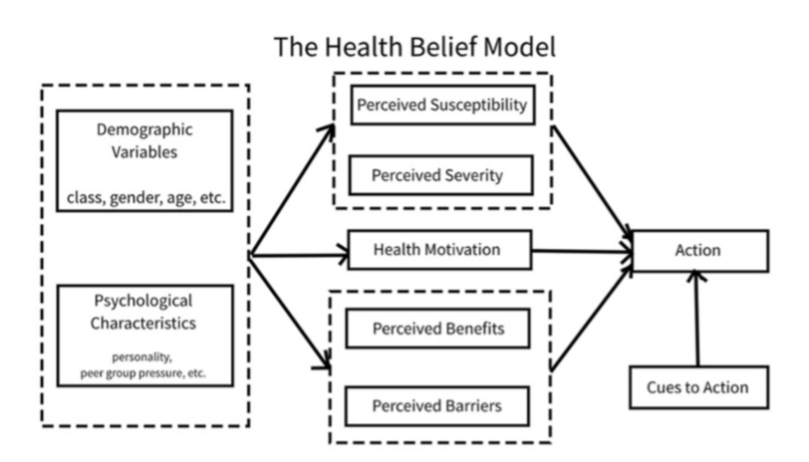
The health belief model of behavior change (reproduced from Fanwang0912 on Wikipedia [24] published under Creative Commons Attribution 4.0 International License [20]).

The themes emerging from previous studies and the literature were aligned with the HBM [23] (Figure 2 [24]). This model suggests that an individual’s likelihood of adopting paraben-free behaviors is based on the individual’s perceived severity of disease, perceived susceptibility to developing diseases related to parabens, and perceived benefits of adopting the health behavior of using paraben-free PCPs [23].

This framework was selected as it emphasizes the psychological and social factors that shape health behaviors. It also provides guidance for designing educational health interventions as it highlights determinants of behavior change. Furthermore, research has underscored how there are positive associations between high knowledge, risk perceptions, and health beliefs and avoidance of lead, parabens, BPA, and phthalates [16].

Although a range of behavior change theories and frameworks, such as the capability, opportunity, and motivation–behavior framework; social cognitive theory; and the social ecological framework, offer valuable perspectives on the social and contextual determinants of behavior, this paper intentionally focuses on the HBM as the primary theoretical foundation for this project. This decision was guided by the aims of the toolkit, which center on addressing individual-level perceptions, decision-making processes, and motivational factors related to paraben-free behavior. Incorporating multiple frameworks risked diluting conceptual clarity and complicating the structure of the toolkit without clear added benefit for this specific application.

### Steps 4 and 5: Ideate Implementation Strategies and Prototype Potential Product

The research team summarized the data from previous steps and developed solutions to improve the toolkit. On the basis of these findings, we identified Wix as a suitable platform that provides tools for creating websites and mobile apps. Through this platform, the Paraben-Free & Me educational toolkit was created in the format of a mobile app. This platform was selected because it could be accessed anywhere and contained blog posts, forums, question-and-answer sections, video and podcast recommendations, and quizzes.

### Step 6: Gather User Feedback on the Prototype

Three users provided feedback to improve our toolkit. Feedback included design suggestions, such as including drop-down menus for blog posts and using smaller paragraphs. In terms of content, the users suggested that podcasts be included, as well as a larger number of videos. Suggestions were also provided on how often to update the toolkit, ranging from every 3 days to including a weekly summary message on Fridays.

### Step 7: Build a Minimum Viable Product

**Figure 3.**
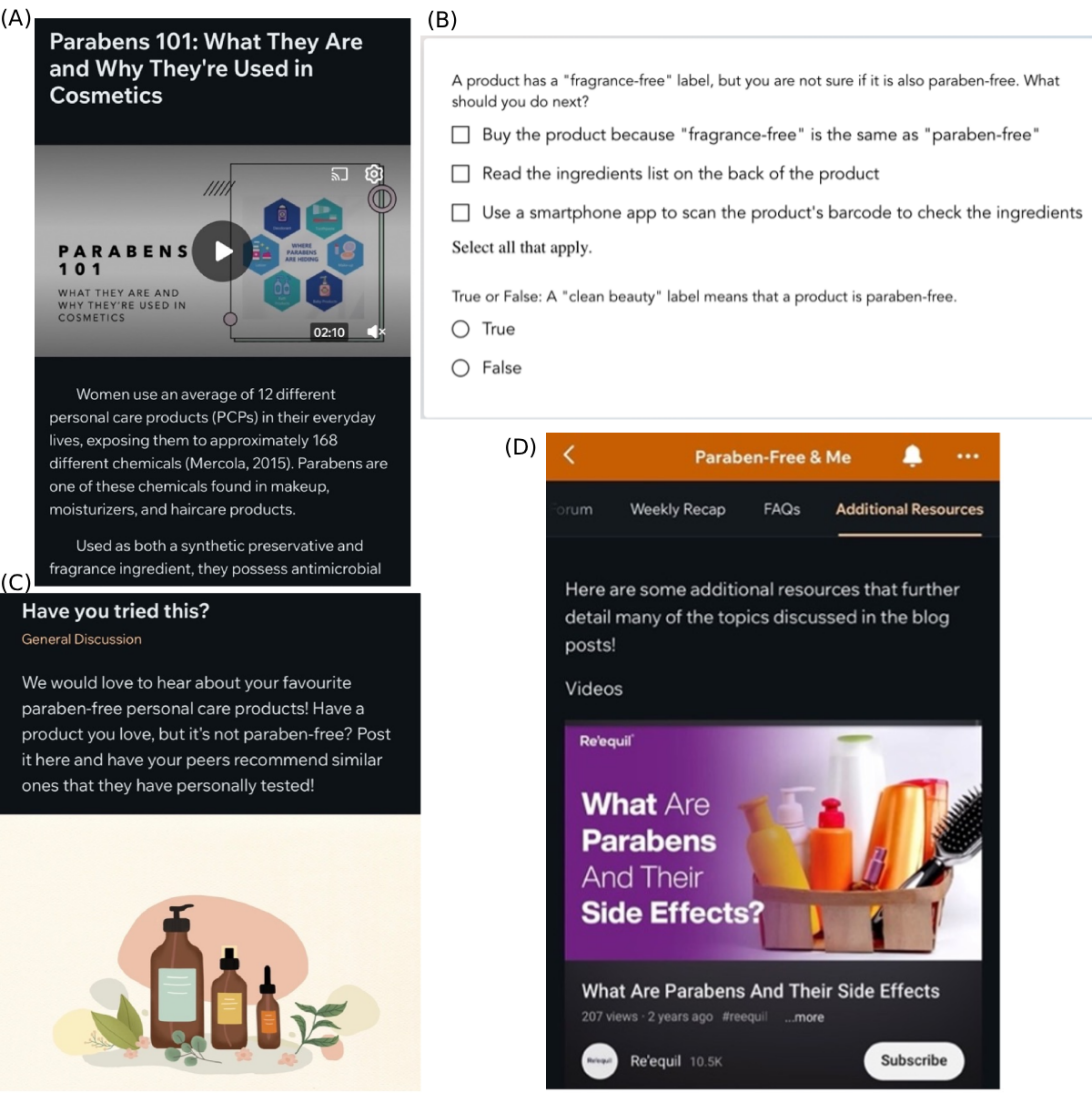
(A) Example blog post featured in the Paraben-Free & Me toolkit to educate users on parabens, their health impacts, and how to avoid them. (B) Example of the weekly quizzes shared with users to test their knowledge and understanding of the blog post material. (C) Example forum page for users to share their knowledge and product recommendations. (D) One of the additional resources that users can access to support their learning.

Through this design process, we built the Paraben-Free & Me toolkit, which included synthesizing previous literature, conducted by members of our research team, and sharing the first version of the toolkit with our research team (Table 1). The final version of the toolkit provides individuals with an array of resources to support learning. These resources include blog posts, quizzes, videos, and podcasts. We ensured that the posts had simple and consistent messaging, features that were highlighted in our systematic review, and incorporated constructs from the HBM (Table 2 and Figure 3A). To ensure that users were able to comprehend the material, we created built-in quizzes for users to test their knowledge on parabens and see what topics they may need to do more reading on (Figure 3B). Moreover, to build a sense of community, we also included a forum section for users to share their knowledge and provide them with the opportunity to recommend other resources or products (Figure 3C). Furthermore, additional resources, such as YouTube videos, documentaries, and podcasts, were included to support personalized learning (Figure 3D).

**Table 1.** Identified characteristics, supportive feedback, and toolkit integration.

Theme	Feedback	Toolkit feature
Simple, consistent, and frequent messaging	Smaller paragraphs, buzzwords, and summarizing blog posts into short messages	Blog posts and weekly messages
Accessibility everywhere	—^a^	Mobile app
Health- and appearance-based motivators	—	Blog posts
Interactivity	Include checkpoints to interact with users	Forum page
Multimedia	Include audio and visual components	Blog posts
Personalization	Include audio and visual components and include additional resources to support different learning styles	YouTube videos, documentaries, and podcast recommendations
Incentives	—	Weekly quizzes

a Theme not discussed in the pilot-testing focus group.

**Table 2.** Paraben-Free & Me intervention design.

Focus of instruction	Learning goal	HBM^a^ construct
“Parabens 101: What They Are and Why They’re Used in Cosmetics?”	Knowledge	Perceived severity and susceptibility
“The Truth About Parabens: What’s the Worst They Can Do?”	Risk perceptions	Perceived severity and susceptibility
“Can Switching to Paraben-Free Personal Care Products Improve Your Health?”	Health beliefs	Perceived benefits
“Are You Sure of What You’re Putting on Your Skin? Learn How to Check the Ingredients of Your Personal Care Products”	Knowledge	Perceived barriers
“How to Decode and Understand Product Labels and Websites for Healthier Choices”	Knowledge	Perceived barriers
“How to Identify Paraben-Free Personal Care Products and Make the Switch: Tips and Tricks for Healthier Shopping”	Paraben-free PCP^b^ behavior	Perceived barriers

a HBM: health belief model.

b PCP: personal care product.

## Discussion

### Principal Findings

Overall, the main purpose of this manuscript was to describe a case study of the application of the IDEAS framework to guide the process of developing a new toolkit designed to promote paraben-free behavior and increase women’s knowledge of parabens. Using the IDEAS framework allowed us to use an iterative design process that ensured that target users were able to provide feedback throughout the process. This ensured that the toolkit would work for the target population. The results from steps 1 and 6 provided important insights into the needs and preferences of the target population. These insights allowed the research team to make corrections and changes to the interactive toolkit during the design phase. In particular, gathering user feedback on the prototype (step 6) was extremely valuable as we were able to make modifications to ensure that the minimum viable product provided the target population with an array of resources.

### Comparison With Prior Work

Through the use of the IDEAS framework, we designed a toolkit that includes several desired features that are consistent with previous research focusing on toolkits. For example, in the focus group conducted to pilot-test the app, the use of videos and audio recordings was suggested to supplement the material presented in the blog posts to provide users with multimedia tools that may assist them in personalizing their learning. This suggestion is consistent with previous literature in which multimedia approaches and personalization of learning were used to increase knowledge and facilitate behavior change [21,22,25,26]. Additionally, the focus group highlighted the need for smaller paragraphs and summary messages at the end of the week. Simple, consistent, and frequent messaging is a common theme among educational toolkits in the literature [21,22,27].

### Future Directions and Implications

Following the IDEAS framework, the next step for this project will be assessing the effectiveness of the Paraben-Free & Me toolkit using a randomized controlled approach. This includes piloting the toolkit for potential efficacy and usability (step 8), evaluating the efficacy through a randomized controlled trial (step 9), and sharing the intervention and findings (step 10). A single-site study at Ontario Tech University will be conducted to measure the effectiveness of the toolkit among female students between the ages of 18 and 35 years, with half of the participants in the control group and half in the intervention group. The primary outcome of assessment will be paraben-free behavior, and secondary outcomes will include knowledge and access to information, health beliefs, and risk perceptions. Both groups will complete the questionnaire that assesses these outcomes at baseline and after the intervention period. Group mean differences and 95% CIs will be analyzed to determine whether there were differences between baseline and follow-up scores. Future iterations of the toolkit may include developing simple games, replacing weekly quizzes with daily morning quotes, and potentially adding incentives through gift vouchers or promotional codes for paraben-free products.

### Limitations

Despite the strengths of the project, there are several weaknesses as well. First, we only had 3 users, all of whom were highly educated, provide feedback on our prototype. If we had had a larger amount of feedback from a more diverse group of women (such as women who are immigrants or not proficient in using technology), our toolkit might have improved as we would have been able to incorporate feedback from a broader range of target users. Finally, pilot testing was not completed for usability and satisfaction. Conducting a small-scale evaluation of the potential efficacy and user satisfaction would have assisted in further refining the toolkit.

### Conclusions

This manuscript described the use of the IDEAS framework to guide the development of the Paraben-Free & Me toolkit, an educational resource aimed at increasing women’s knowledge and paraben-free behaviors. A scoping and systematic review was used to ensure that the appropriate characteristics were included, with feedback from the research team used to further support these findings and ensure their proper use within the development of the toolkit. The IDEAS framework provided a method for focusing on a human-centered and iterative development process to ensure that the toolkit was evidence-based and its efficacy could be evaluated. The Paraben-Free & Me toolkit has the potential to help overcome barriers faced by women from diverse backgrounds as it clearly presents evidence on the harms associated with parabens and provides practical guidance for identifying them in PCPs. By minimizing paraben exposure, the toolkit promotes better health outcomes and improved quality of life.

## Abbreviations

BPA: bisphenol A
EDC: endocrine-disrupting chemical
HBM: health belief model
IDEAS: integrate, design, assess, and share
PCP: personal care product
